# A customizable aluminum compensator system for total body irradiation

**DOI:** 10.1002/acm2.13393

**Published:** 2021-08-25

**Authors:** Madison Naessig, Soleil Hernandez, Nestor Rodrigo Astorga, James McCulloch, Daniel Saenz, Pamela Myers, Karl Rasmussen, Sotirios Stathakis, Chul S. Ha, Niko Papanikolaou, John Ford, Neil Kirby

**Affiliations:** ^1^ Department of Radiation Oncology The University of Texas Health Science Center at San Antonio San Antonio Texas USA; ^2^ Department of Nuclear Engineering Texas A&M University College Station Texas USA

**Keywords:** compensator, dose homogeneity, total body irradiation

## Abstract

**Purpose:**

To develop a simplified aluminum compensator system for total body irradiation (TBI) that is easy to assemble and modify in a short period of time for customized patient treatments.

**Methods:**

The compensator is composed of a combination of 0.3 cm thick aluminum bars, two aluminum T‐tracks, spacers, and metal bolts. The system is mounted onto a plexiglass block tray. The design consists of 11 fixed sectors spanning from the patient's head to feet. The outermost sectors utilize 7.6 cm wide aluminum bars, while the remaining sectors use 2.5 cm wide aluminum bars. There is a magnification factor of 5 from the compensator to the patient treatment plane. Each bar of aluminum is interconnected at each adjacent sector with a tongue and groove arrangement and fastened to the T‐track using a metal washer, bolt, and nut. Inter‐bar leakage of the compensator was tested using a water tank and diode. End‐to‐end measurements were performed with an ion chamber in a solid water phantom and also with a RANDO phantom using internal and external optically stimulated luminescent detectors (OSLDs). In‐vivo patient measurements from the first 20 patients treated with this aluminum compensator were compared to those from 20 patients treated with our previously used lead compensator system.

**Results:**

The compensator assembly time was reduced to 20–30 min compared to the 2–4 h it would take with the previous lead design. All end‐to‐end measurements were within 10% of that expected. The median absolute in‐vivo error for the aluminum compensator was 3.7%, with 93.8% of measurements being within 10% of that expected. The median error for the lead compensator system was 5.3%, with 85.1% being within 10% of that expected.

**Conclusion:**

This design has become the standard compensator at our clinic. It allows for quick assembly and customization along with meeting the Task Group 29 recommendations for dose uniformity.

## 1 INTRODUCTION

Total body irradiation (TBI) is a form of external radiotherapy used to treat various hematopoietic cancers by irradiating the patient's whole body to immunosuppress the bone marrow and kill tumor cells allowing the patient to receive a bone marrow transplantation.^1^, ^2^, ^3^ The overall dosimetric goal of TBI is to treat the whole body within ±10% of the prescribed dose.^4^ The variations in patient separation across different body regions ultimately requires modulation of the radiation fluence to achieve this uniformity goal. Various techniques have been created and implemented into clinics for this purpose. A translational couch method achieves a homogenous dose by varying the velocity of the couch while dose is being delivered by a standard linac to the patient in supine/prone position.^5^, ^6^, ^7^, ^8^, ^9^ For other techniques, the patient stays stationary while an arc sweeps along them.^10^, ^11^, ^12^ Volumetric modulated arc therapy (VMAT) is another technique utilized to achieve a homogenous whole‐body dose.^13^, ^14^, ^15^, ^16^, ^17^, ^18^ Total marrow irradiation is an alternate technique for preconditioning a patient for a bone marrow transplant.^17^, ^18^ This technique instead targets the marrow, which allows it to reduce dose to normal organs.

There is information suggesting that delivering TBI treatment with a low dose rate can reduce the risk of pneumonitis.^16^, ^19^, ^20^ Therefore, a large number of clinics deliver TBI at dose rates at or below 10–15 cGy/min. This requires long irradiation times for patients even with large open‐field techniques. Although beam modulation approaches improve dose uniformity, they can result in significantly longer treatment times, typically on the order of an hour or more. This is caused by the fact that many of these techniques mostly only treat small fraction of the body at a given time. These longer treatment delivery times can result in patient discomfort and are problematic for vault schedules. The extended source‐to‐surface distance (SSD) field‐in‐field technique is an exception to this.^21^, ^22^ For this technique, the full body can be contained in the field and can be implemented in a way to maintain small treatment times. Blood circulation is another critical aspect to consider for these modulated TBI approaches. Although the modulated approaches aim to deliver a uniform dose to the body, circulating cells can have a significantly higher dose heterogeneity than those that are stationary.^23^, ^24^ The factors that contribute to this dose heterogeneity are short treatment times (<20 min), high dose rates, long perfusion periods, and low fraction number regimens.

Although there is an increasing implementation of these modulated beam approaches, the most common TBI technique is to deliver this with large open beams to the patient at extended distances from the source.^25^ The use of tissue compensators is a standard‐of‐care technique for improving dose uniformity in this setup.^25^, ^26^ A compensator is created using a patient's position and measured dimensions at multiple segments along the body. The thickness of the compensator at each segment is then determined based on the SSD and thickness of the corresponding body segment.^17^ The varying thickness of the compensator at each segment attenuates the beam to produce a uniform dose distribution along the central long axis of the patient.

Previously, we constructed a compensator system that was sectioned by regions of interest using lead pieces that varied in thickness and width. These pieces were custom cut for each part of the anatomy and then fixed (taped/bolted) to a compensator tray. This technique required a great attention to detail, which could potentially lead to inaccuracies. The process of designing and constructing a lead compensator for TBI treatments took 2–4 h for a medical physicist to complete at our cancer center. Also, lead was not an easy material to work with and posed a safety concern due to the amount of time a physicist was in contact with it. To develop a simplified and easily customizable compensator system, we propose the implementation of aluminum as the attenuating material and a design that is more efficient in shortening assembly time.

## 2 MATERIALS AND METHODS

### 2.1 Compensator design

**FIGURE 1:**
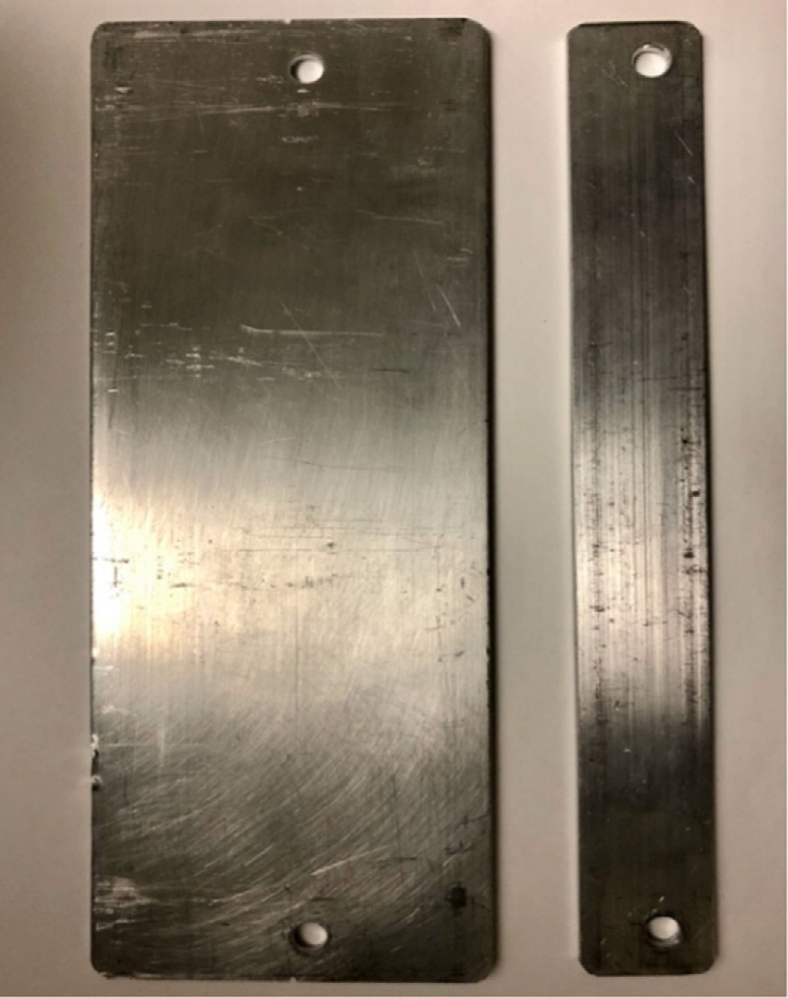
An image of the fixed width (7.6 and 2.5 cm) of the individual aluminum bars that make up the compensator. These bars are all 21.8 cm in length. The holes are offset to the right of the middle of these bars to produce a tongue and groove effect when assembled

The newly designed compensator is composed of 0.3 cm (1/8″) thick aluminum bars with two fixed widths, 7.6 cm and 2.5 cm (3″ and 1″). Compensation throughout the body is performed with a quantized number of these bars stacked in front of anatomical regions. The thickness was chosen as an optimal balance between accuracy and practicality. These bars each produce an attenuation of 3.5%. This quantized amount of attenuation allows for the creation of compensated dose output in each sector that is within 1.75% of that desired. Thinner bars would require more pieces and take longer to assemble. Thicker bars would allow less control of compensator accuracy. Aluminum was chosen due to its ease of machinability and its mechanical strength. More specifically, other metals with a thickness that produces 3.5% attenuation would be easy to bend and not practical for long‐term usage. The two fixed bar widths (7.6 and 2.5 cm) were chosen to be the simplest possible representation of the widths used in our previous lead compensator design (see Figure 1). The more different types of pieces would increase the assembly complexity. Using these two fixed widths provides a reasonable approximation of the previous custom width compensator.

**FIGURE 2:**
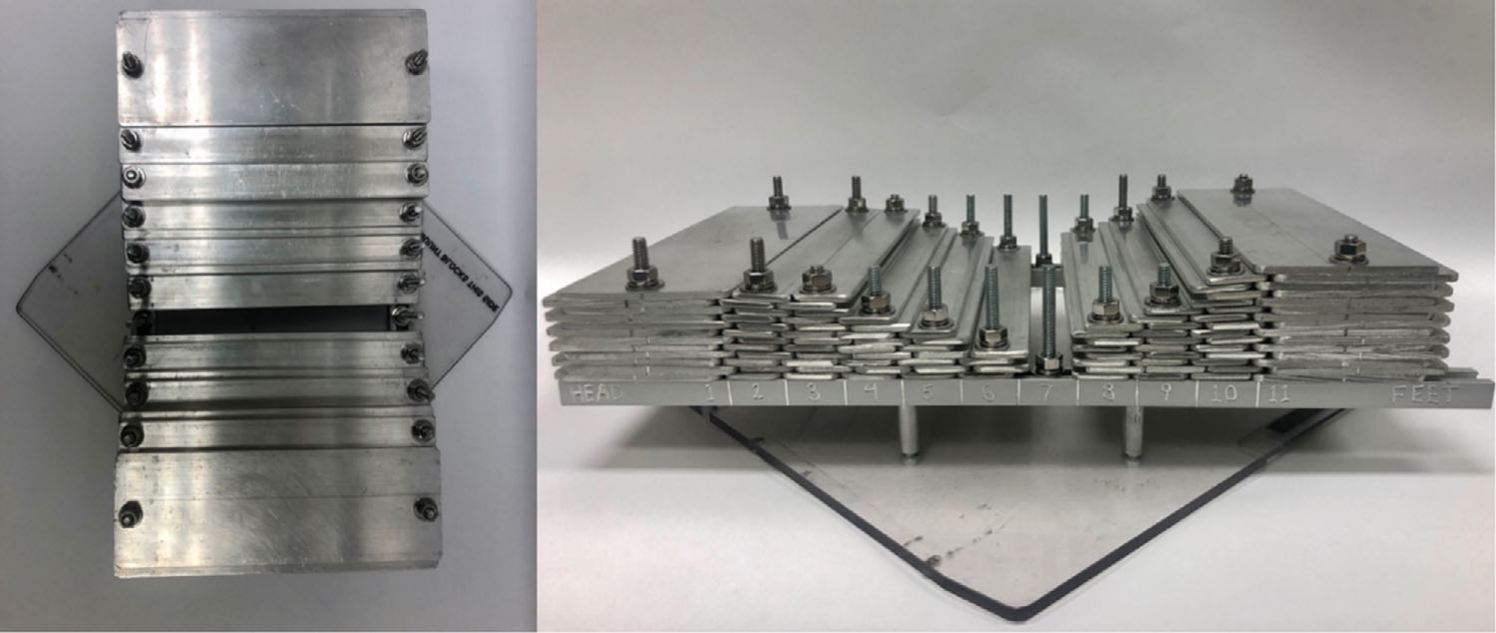
Images of a constructed aluminum compensator from a beam's‐eye view (left) and from the side (right). The various sectors utilizing different sized bars along with the method used to secure the aluminum bars down to the T‐tracks is illustrated. The image also displays the tongue and groove arrangement between each sector that is used to minimize inter‐bar transmission

The aluminum bars are secured to aluminum T‐tracks using metal ¼”‐20 bolts, washers, and hex nuts. These T‐tracks are mounted onto a plexiglass block tray using 10–32 (unified thread standard) bolts and spacers, which then allows the entire system to be inserted into the head of the gantry. The T‐tracks and bolts are spaced far enough apart so that the attenuation they produce is outside of the patient anatomy. Thus, they do not affect the patient dose distribution. The compensator is divided into 11 sectors spanning from the patient's head to feet with the outermost sectors (1 and 11) utilizing the 7.6 cm wide aluminum bars and the innermost sectors (2–10) utilizing the 2.5 cm wide bars. The aluminum bars consist of offset holes at each end. Each piece is assembled with alternating offset direction. This allows them to be interconnected at each adjacent sector with a tongue and groove configuration, which is shown in Figure 2. A typical mass for a fully assembled compensator tray is 7 kg.

### 2.2 Patient setups

**FIGURE 3:**
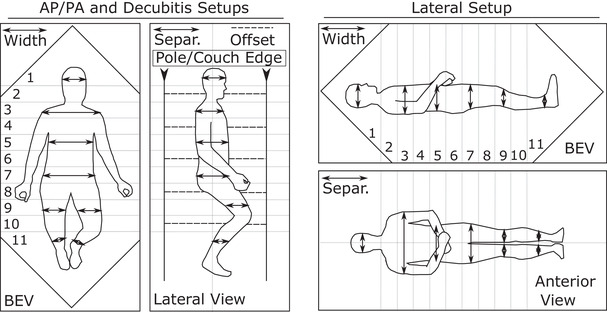
A schematic showing the beam's eye view (BEV) and a view orthogonal to this in various positional techniques used for TBI treatments. Both the width and separation (separ.) are utilized for dose calculation. The approximate cutoff for each sector (1–11) along the length of the patient's body is outlined for both setups

The compensator system is used for anteroposterior‐posteroanterior (AP/PA), lateral, and decubitus treatment setups. For the first of these, patients are treated with AP/PA beams while half seated on a bicycle seat in a TBI stand. Secondly, a lateral setup, involves two parallel opposed lateral fields with the patient lying supine on a bed. For decubitus treatments, the patients are treated with AP/PA beams while they lie on their side in a bed. With taller patients, the knees will be bent so that the patient can fit properly in the field on the bed. The lateral and decubitus setups are often utilized with pediatric patients or patients that are unable to stand for long periods of time. Lung blocking is not part of the aluminum compensator system. Separate Cerrobend lung blocks are created for patients that require them. A diagram of each positional technique is shown in Figure 3. The alignment of the compensator relative to the anatomy is recorded during each simulation. As an example, a typical AP/PA setup places the axilla at the transition between sectors 3 and 4. For lateral setups, the neck‐shoulder transition is always set up to the line between two sectors (most commonly 1 and 2). This is critical for this setup as it enables the compensator to rapidly change aluminum thickness at the same place that there is a large variation in the midplane tissue depth. Beyond recording the compensator alignment, the separation and width of the patient is manually measured at the middle of each sector with body calipers.

### 2.3 Compensator calculation

For TBI treatments, there are various dose calculations that are necessary to compute before the treatment begins. Specifically, a planning calculation must be used to construct and customize the compensator correctly for each individual patient. The following equation is the planning calculation that has been implemented, which determines the compensator thickness as well as the monitor units required for a particular treatment:

(1) $$\begin{array}{c} \left(\frac{Dose}{MU}\right)={\left(\frac{Dose}{MU}\right)}_{reference}\;\cdot OAF\left(Sector\right)\cdot TPR\left(Depth\right)\\ \cdot CF\left(Width,\;Separation\right)\cdot CT\left(Compens.\;\#\right)\cdot {\left(\frac{340}{SMD}\right)}^{2} \end{array}$$

In this equation *OAF* is the off‐axis factor for a particular sector, *TPR* is the tissue phantom ratio relative to 10 cm depth, *CF* is the correction factor for phantom scatter multiplied by *TPR* when accounting for patient width and separation, *CT* is the compensator transmission, and *SMD* is the source to midplane distance. The dose per MU is calculated for a combination of opposed beams. The reference conditions consist of a 30 × 30 × 20 cm phantom (20 is along the beam direction), at a SMD of 340 cm, and a 10 cm depth with a spoiler and tray employed. These compensators are made such that the same compensator is used for both treatment directions. The TPR data utilized here is one dimensional, representing the attenuation along the central beam axis. TPR values change off this central axis. The importance of this variation was explored. In brief, these deviations were less than 3% for the vast majority of the typical clinical combinations of midplane depths and sectors. Beyond this, none of these combinations were found that would cause midplane dose errors of 10% or greater. However, this is an issue that all institutions should evaluate when implementing their own calculations.

For patient calculation, we calculate the uncompensated dose rate at each of the 11 sectors. The MU set for the patient is based on the sector with the lowest dose rate. The dose rates for all of the sectors are then normalized by that of the lowest dose rate section yielding the dose the sectors would receive relative to the prescription without compensation. The inverse of this number is used along with the transmission data to interpolate the number of compensators needed for each individual sector.

### 2.4 Inter‐bar transmission

With the bars being offset at each adjacent sector, a tongue and groove effect is created. To verify the resulting transmission between the aluminum bars, a water tank and diode setup was assembled. These measurements were performed with a PTW BEAMSCAN and a PTW 60012 Diode E, using a 1 mm scanning resolution. More specifically, the water tank measurements were made at 100 cm SSD, 1.5 cm depth, and an open 40 × 40 cm field with the aluminum compensator in place.

### 2.5 Commissioning measurements

To commission this compensator system in our clinic, it was necessary to obtain the various factors in Equation (1). A brief description of this follows. The value of ${\left(\frac{Dose}{MU}\right)}_{reference}$ is obtained through a ratio of ion chamber (0.3 cc PTW Semiflex) charge measurements in the reference setup to one with a known *Dose*/*MU* at isocenter. Except for this isocenter measurement, all measurements occur at extended SSD, with a spoiler and tray in place for a 40 × 40 cm field. Values for OAF are obtained through charge measurements from an ion chamber inside the 30 × 30 × 20 cm phantom at a 10 cm depth in each of the 11 sectors. The ratios of these charges to that on the central axis are the OAFs for each corresponding sector. Values for TPR are determined with charge measurements from varying depths in solid water at 340 cm SMD in the TBI setup. These TPR measurements all have a phantom size of 30 × 30 cm transverse to the beam direction. To correct these for the patient contour and scatter differences caused by different patient widths, calculations were performed using Adaptive Convolve in the Pinnacle treatment planning system (version 16.2). For these calculations, cylindrical density overrides were created. These cylinders were 1 m long in the superior/inferior direction and were elliptical in the axial cross section. Elliptical density overrides were created to cover the range of patient widths and separations. The Pinnacle calculated midplane dose for these elliptical density overrides over the dose for a solid water stack with the same separation was used to determine a scatter correction factor (CF). Held et al. have demonstrated that the Pinnacle treatment planning system is accurate for extended‐SMD TBI calculations.^12^ The scatter correction was tabulated as a function of width and separation for patient calculation. Bi‐linear interpolation is utilized to determine a value of CF from these data for the measured patient width and separation at each sector. The CT values were determined through charge measurements obtained as a function of the number of aluminum bars attenuating the beam.

### 2.6 End‐to‐end validation

End‐to‐end measurements were performed using a RANDO phantom with internal and external OSLDs as well as PTW Semiflex 0.3 cc ion chamber in a 30 × 30 × 20 cm solid water phantom. This solid water phantom does not match the true scatter conditions of the TBI calculations but is included to be additional information to supplement the RANDO measurements. The RANDO phantom included the head, thorax, abdomen, and lower torso but did not include the legs. The RANDO phantom was treated in two treatment positions, lateral and decubitus. These were aligned to the compensator relative to the transition between sectors 3 and 4. More specifically, the lateral and decubitus setups were positioned with the neck‐shoulder transition and axilla, respectively, at this location. An aluminum compensator was constructed based on the calculation in Equation (1) and the measured RANDO width and separation, and inserted into the gantry head. OSLDs were positioned on the surface (under 5 mm of bolus) of the RANDO phantom at sectors 2 through 9 as well as on the inside of the RANDO phantom (at midplane) for sectors 2,4,6, and 8. For both setups, sectors 2, 4, 6, and 8 were aligned to the head, shoulder, umbilicus, and pelvis of the phantom, respectively. The surface measurements were made with the OSLDs placed in the middle of the sectors. In contrast, the internal measurements were placed between RANDO phantom slabs, which were not always at the middle of the sectors. This resulted in a total of 24 point validation measurements. The percent error between the planned and measured dose was calculated for each OSLD measurement. The RANDO phantom was not treated in an AP/PA setup due to safety concerns of the phantom falling from an upright position.

The solid water was treated in AP/PA and lateral treatment positions. For the lateral setup, the solid water was translated and treated at the location of each of the sectors. With the AP/PA setup, the solid water stack was supported with straps and moved vertically and treated at each sector location. A total of 22 ion chamber measurements were documented and the percent error between the planned and measured dose was calculated. The planned dose was calculated with Equation (1) from the measured phantom width, separation, SMD, and compensator thickness at each sector. Surface and internal measurements used TPR values representative of that under the bolus and at midplane, respectively.

### 2.7 In‐vivo dosimetry

In‐vivo dosimetry was performed for each of our patients undergoing treatment. This involved OSLDs being placed on the patient underneath 5 mm of bolus for both beam directions. However, for the lung block in‐vivo measurements, the OSLDs needed to be placed under 10 mm of bolus due to electron contamination.^21^, ^27^ The expected dose on the surface of the patient was calculated. The results for 20 patients were collected and analyzed. These results were also compared against those for 20 patients with a prior lead compensator.

To further evaluate the performance of the aluminum compensator compared to the lead compensator, a statistical analysis was performed. The in‐vivo dosimetry measurements for 40 patients were compared, 20 patients with the aluminum compensator in place and 20 patients with the lead compensator in place. A Shapiro–Wilk test was completed to determine the normality between both sets of measurements as well as Wilcoxon Rank Sum Test.

### 2.8 Average patient compensators

Sixty TBI patients (53 AP/PA and 7 lateral) were treated with this compensator system during the first year of use. The compensator appearances for these two different setups are quite different. AP/PA filters vary slowly from one sector to the next, but the lateral filters have a large gradient at the neck‐shoulder transition. The average, minimum, maximum, and standard deviation for the number of compensator bars in each sector was recorded for each of the setups. This information is useful in showing the importance of using patient specific TBI compensators. Additionally, extended SSD water tank scans were performed along the length of the average lateral compensator to determine the dose gradients produced from this device and the resulting need for positional accuracy. More specifically, the linac was rotated laterally to the same angle used for lateral TBI patients. Then, the water tank was positioned in the location of TBI treatments and moved as far from the linac as possible, which produced a 328 cm SSD. The water tank was filled with a 30 cm depth of water and a PTW 31010 ionization chamber was positioned in the center of this at a 15 cm depth. The chamber was kept at this water depth, with 10 cm of buildup along the beam direction, and scanned from the head to foot direction of a lateral TBI patient. Thus, the produced measurements represent those at 10 cm beam depth for a patient with a 30 cm scatter size. The average lateral patient had a maximum difference of five bars between adjacent sectors (11 for sector 1 and 6 for sector 2). In addition to the average compensator, we performed an additional scan with a difference of 11 bars between sectors 1 and 2. This is a typical difference in the number of compensators between the shoulder and neck sectors.

## 3 RESULTS

**FIGURE 4:**
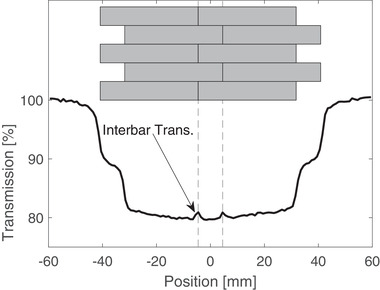
Beam profile for a 6 MV radiation beam with aluminum compensator placed in the pathway of the beam to illustrate the inter‐bar transmission

The resulting transmission between the offset aluminum bars was analyzed by PTW MEDPHYSTO software. Figure 4 displays the beam profile that was measured with a 6 MV beam and a PTW diode E. The inter‐bar transmission deviates by 2% at the greatest difference.

**FIGURE 5:**
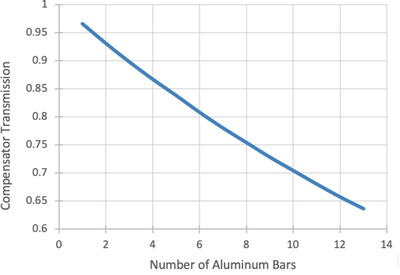
Compensator transmission results as multiple aluminum bars are stacked and secured on top of each other in the path of the radiation beam

Figure 5 displays the compensator transmission versus the number of aluminum bars that are used to construct a specific sector within the compensator. The transmission decreases with each individual aluminum bar that is placed in the radiation beam's pathway. The first aluminum bar caused a decrease in compensator transmission of 3.5%. When 13 aluminum bars were secured to one another the measured transmission resulted in a decrease of 36.4%.

**FIGURE 6:**
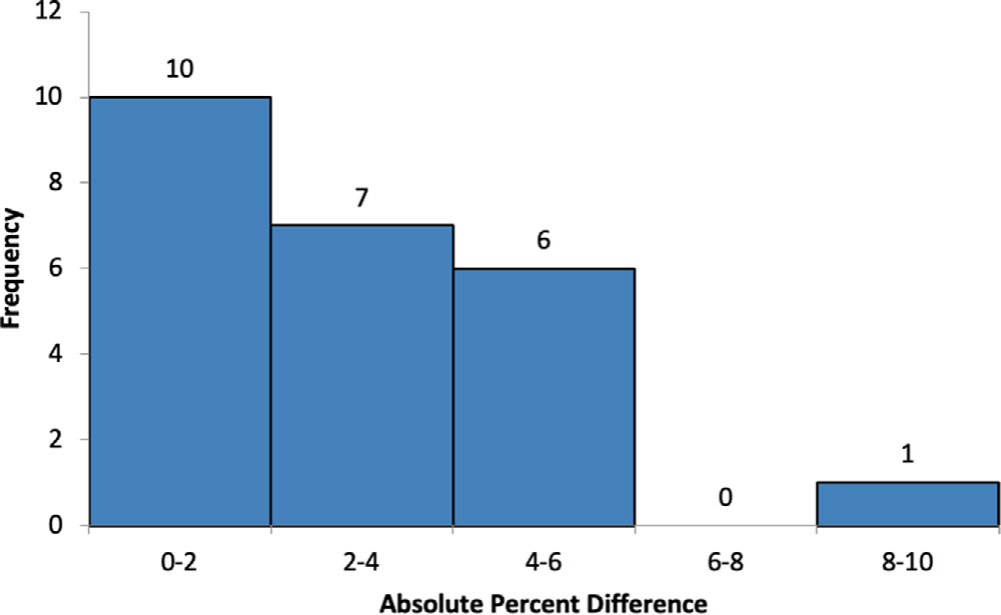
The end‐to‐end validation results for the RANDO phantom positioned in two different positions, lateral and decubitus (see Section 2.6). The absolute percent difference between planned and measured dose for each sector was calculated and plotted

**FIGURE 7:**
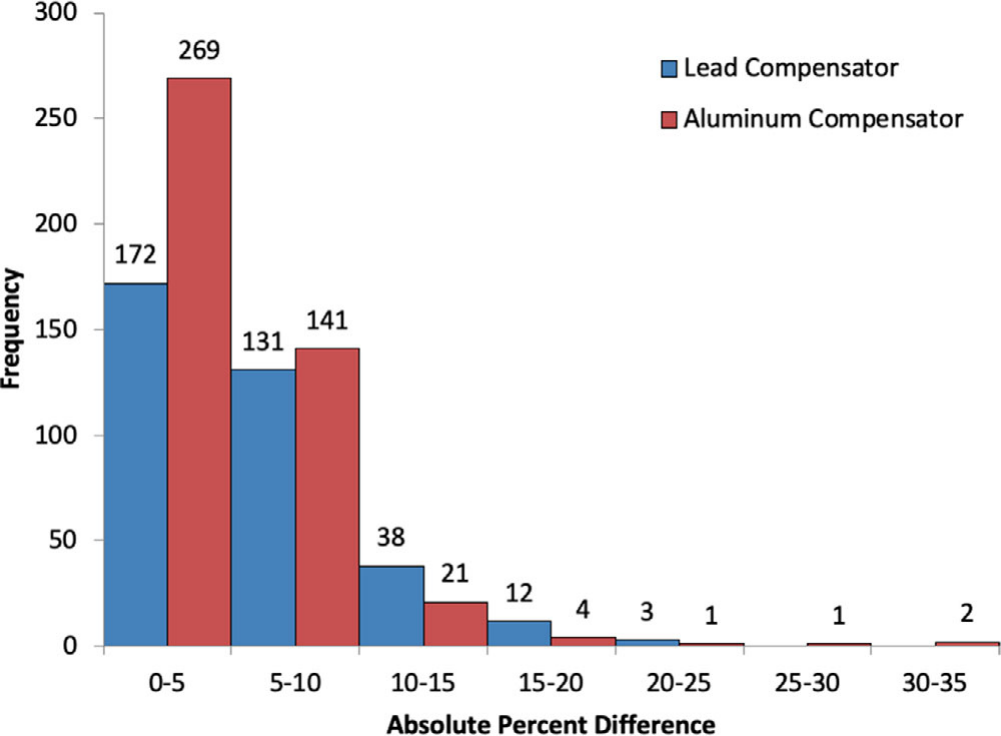
The in‐vivo dosimetry measurements for 40 patients utilizing both the lead and aluminum compensator. The absolute percent difference between planned and measured dose for each sector was calculated

The solid water end‐to‐end validation measurements had a mean error of 1.4%, with a maximum deviation of 3.2%. The OSLD end‐to‐end validation and in‐vivo dosimetry results are displayed in Figures 6 and 7, respectively. Figure 6 shows all end‐to‐end OSLD measurements were within ± 0%. For the in‐vivo measurements, an OSLD was placed on each region of the patient both anteriorly and posteriorly correlating to each sector of the compensator, resulting in 22 OSLD measurements for each patient. These results are displayed for the original lead compensator and the new aluminum compensator system. The absolute percent differences between planned and measured were calculated and recorded. All in‐vivo measurement deviations greater than 10% are investigated further and discussed in the medical physics consultation. The responsible therapist is questioned about the possibility for misplacement and if the OSLD fell off during treatment. If no issues are found from the therapist, the deviations are compared to those for adjacent sectors with similar shielding and SSD values. If the patient is receiving multiple fractions, a new measurement is performed. Otherwise, the information from the investigation is used to determine whether the deviation is a true reflection of the dose the patient received in the measured sector. None of the aluminum compensator deviations greater than 15% were found to be real. We do not have as much information on the deviation causes for the historical lead compensator to screen that data. For this reason, all in‐vivo data are shown and analyzed here.

The median absolute error produced by the aluminum and lead compensators were 3.7% and 5.3%, respectively. A Shapiro–Wilk normality test demonstrated the absolute percent error between the planned dose and measured dose for both the aluminum compensator and lead compensator was not normally distributed. Therefore, a Wilcoxon Rank Sum Test was performed, with a null hypothesis that there is no difference between the aluminum and lead median absolute errors. The *p*‐value produced from this was 5 × 10^–7^, suggesting that we must reject the null hypothesis and that there is a significant difference between the medians. Beyond this, 93.8% of the aluminum compensator OSLD measurements were within ±10% of the expected values. This number was only 85.1% with the previous lead compensator.

Tables 1 and 2 display the variations in the compensators for the first year of operation. The dose gradient and need for positional accuracy is controlled by the difference in the number of compensators between adjacent sectors. The maximum measured dose gradient for the average lateral compensator (with a difference of five bars between sectors 1 and 2) was 3.5% per cm. The additional scan with an 11 bar difference between sectors 1 and 2 produced a maximum dose gradient of 7.8% per cm.

**TABLE 1:** Variations for AP/PA patient TBI compensators

Sector	Anatomy mode	Average	Standard deviation	Minimum	Maximum
1	Head	6.7	2.4	1	12
2	Head	5.2	2.2	1	10
3	Shoulder	4.9	2.3	0	9
4	Chest	4.2	2	1	9
5	Umbilicus	2.4	1.6	0	7
6	Umbilicus	0.8	1.3	0	6
7	Pelvis	2.2	2	0	9
8	Thigh	5.2	2.4	0	12
9	Knee	5.9	2.4	0	12
10	Lower leg	6.3	2.2	1	11
11	Foot	5.5	2.2	1	11

*Note*: Each sector is labeled with the most common anatomy and the average, standard deviation, minimum, and maximum number of compensator bars during the first year of operation.

Abbreviations: AP/PA, anteroposterior‐posteroanterior; TBI, total body irradiation.

**TABLE 2:** Variations for lateral patient TBI compensators

Sector	Anatomy mode	Average	Standard deviation	Minimum	Maximum
1	Head	10.7	2.7	7	14
2	Shoulder	5.6	7	0	15
3	Chest	2.9	2.5	0	6
4	Chest	3.9	2.6	0	7
5	Pelvis	3.4	3.1	0	9
6	Pelvis	2.6	3	0	8
7	Thigh	5.1	2	2	8
8	Knee	7.6	3.6	4	15
9	Lower leg	8.7	2.6	5	13
10	Lower leg	10.4	2.3	7	14
11	Foot	10	2.1	7	13

*Note*: Each sector is labeled with the most common anatomy and the average, standard deviation, minimum, and maximum number of compensator bars during the first year of operation.

Abbreviations: TBI, total body irradiation.

## 4 DISCUSSION

The time required to construct the aluminum compensator has been reduced to 20–30 min compared to the 2–4 h it would take with the previous lead design. This difference in time has allowed the medical physics staff and residents to better manage and allocate their time to other responsibilities throughout the workday. The technique has limitations. More specifically, it is only a one‐dimensional compensation technique that produces a uniform dose distribution along the central long axis of the patient. It does not ensure uniformity in regions far of the axis, such as the arms. It is also not capable of producing a total marrow irradiation treatment.

Analysis of the inter‐bar diode profile exhibited an increase in transmission at the interconnection of the aluminum bars. However, the magnitude of transmission did not exceed 2%, which is acceptable considering the dosimetric goal is to be within ±10%. Beyond this, the diode measurements at isocenter are more of a worst‐case scenario for this transmission. This is because the measurements are made directly on the central axis, which allows multiple inter‐bar transitions to line up. The aluminum bars stacks do not follow divergence. For parts of the compensator offset from the central axis, subsequent inter‐bar transitions do not line up.

The end‐to‐end measurements were all within ±10%, but the same cannot be said for patient treatment. The largest errors found are believed to be due to OSLD placement issues. However, not all measurements were completely fixed through correct placement. Many of these measurements were corrected through either removing or adding aluminum bars to the individual sector where the OSLD was placed.

The produced dose gradient of the compensator can be summarized as 0.7%/cm per bar difference between adjacent sectors. For the head‐shoulder transitions with lateral patients, achieving 10% accuracy requires positioning accuracy within 1 cm. However, for all other regions, where you typically only get two or three bar differences between adjacent sectors, there is a much lower dosimetric effect caused by positional uncertainty.

The compensator variations in Tables 1 and 2 are useful in evaluating the achievable dosimetric accuracy from having a standard compensator for all patients of a given setup (such as AP/PA or lateral). More specifically, the differences in the number of compensator bars can be directly related to beam attenuation (see Figure 5) and delivered dose differences. Most of the standard deviations are around two to three bars for each sector. Three bars also produce approximately 10% beam attenuation. Thus, with a standard compensator, one would expect in‐vivo measurement errors with a standard deviation of around 10% (or higher given the other measurement uncertainties). In comparison, the compensator system here has 93.8% of measurements within 10%. Therefore, errors greater than 10% are close to a two standard deviation event. Evaluating the mean, maximum, and minimum number of bars shows more of the worst‐case scenarios for dosimetric errors for a standard compensator. It is common for either the minimum or maximum number of bars to be separated by the average by five bars (16% attenuation) or more from the average.

The total number of small aluminum bars fabricated was 180 along with 60 big aluminum bars. These numbers were found through reviewing previous patient measurements, with the intent of being able to support four simultaneous TBI patients. This has, in general, been the case at our clinic. Lateral patients make up 10% of our TBI treatments. These treatments require more compensator bars than AP/PA treatments. Thus, the four simultaneous patient number is reduced when lateral patients are involved.

## 5 CONCLUSION

The speed and accuracy of constructing compensators for TBI treatments has improved with this customizable aluminum design. It allows treatments to meet universal dosimetric goals of planned dose and measured dose being within ±10%. This compensator design is the standard technique currently utilized at our clinic.

## CONFLICT OF INTEREST

The authors declare no conflict of interest.

## AUTHOR CONTRIBUTIONS

All authors have made substantial contributions to the analysis of this work, have helped draft the manuscript, provided approval of the paper, and have worked to ensure the accuracy of the results presented here.

## Data Availability

All the meaningful data are available in the manuscript.
